# Retinol binding protein 4 and risk of type 2 diabetes in Singapore Chinese men and women: a nested case-control study

**DOI:** 10.1186/s12986-018-0329-0

**Published:** 2019-01-10

**Authors:** Yeli Wang, Liang Sun, Xu Lin, Jian-Min Yuan, Woon-Puay Koh, An Pan

**Affiliations:** 1 Health Services and Systems Research, Duke-NUS Medical School, Singapore, 169857 Singapore; 2 CAS Key Laboratory of Nutrition, Metabolism and Food safety, Shanghai Institute of Nutrition and Health, Shanghai Institutes for Biological Sciences, University of Chinese Academy of Sciences, Chinese Academy of Sciences, Shanghai, 200031 China; 3 Division of Cancer Control and Population Sciences, UPMC Hillman Cancer Center, University of Pittsburgh, Pittsburgh, 15232 PA USA; 4 Department of Epidemiology, Graduate School of Public Health, University of Pittsburgh, Pittsburgh, 15261 PA USA; 5 Saw Swee Hock School of Public Health, National University of Singapore and National University Health System, Singapore, 117549 Singapore; 6 Department of Epidemiology and Biostatistics, School of Public Health, Tongji Medical College, Huazhong University of Science and Technology, Wuhan, 430030 Hubei Province China

**Keywords:** Cohort studies, Meta-analysis, Nested case-control study, Retinol binding protein 4, Type 2 diabetes

## Abstract

**Background:**

Although retinol binding protein 4 (RBP4) has been implicated in insulin resistance in experimental studies, the association between RBP4 and risk of type 2 diabetes remains unclear. We assessed this association in a Chinese population, and pooled our results with those from two prior studies.

**Methods:**

Plasma RBP4 levels were measured among 571 incident type 2 diabetes cases and 571 controls nested within the Singapore Chinese Health Study. All participants were free of diabetes, cancer and cardiovascular disease at blood collection (1999–2004). Incident cases of physician-diagnosed diabetes were self-reported at subsequent interviews (2006–2010).

**Results:**

Plasma RBP4 levels were significantly higher in men than women, and the respective median values were 30 (interquartile range: 24–35) μg/mL and 25 (interquartile range: 21–31) μg/mL, respectively. With adjustment for diabetes risk factors, compared to the lowest quartile, the odds ratio (OR) and confidence interval (CI) for risk of type 2 diabetes associated with the highest quartile of RBP4 levels were 1.23 (0.73–2.07; *P*-trend = 0.14) in all subjects, 0.63 (0.27–1.45; *P*-trend = 0.65) in men, and 2.29 (1.05–5.00; *P*-trend = 0.018) in women. The difference in the risk estimates between men and women was statistically significant (*P*-interaction = 0.032). When we pooled our results with two prior studies, ORs (95% CIs) comparing high versus low category of RBP4 was 1.01 (0.70–1.46; *I*^*2*^ = 8.2%; *P*-heterogeneity = 0.34) in men, and 1.73 (1.28–2.33; *I*^*2*^ = 0%; *P*-heterogeneity = 0.80) in women.

**Conclusions:**

Increased plasma RBP4 levels were associated with higher risk of type 2 diabetes in women but not in men.

**Electronic supplementary material:**

The online version of this article (10.1186/s12986-018-0329-0) contains supplementary material, which is available to authorized users.

## Background

Retinol binding protein 4 (RBP4) was originally discovered as a hormone secreted by the liver and acting as a transport protein for vitamin A (retinol) from the liver to peripheral tissues [1]. However, recent research has identified adipocytes as another source of RBP4 [2], and evidence from animal and human studies has implicated the involvement of RBP4 in the pathogenesis of insulin resistance [3] and type 2 diabetes [4, 5]. For example, in murine models, injection of RBP4 in normal mice has been shown to impair muscle insulin signaling, while deletion of *RBP4* gene could enhance insulin sensitivity [3]. In human studies, decreasing blood RBP4 levels by drug therapies improved insulin resistance [3], and a link between *RBP4* gene and type 2 diabetes has been found in genetic studies [4, 5]. In addition, elevated RBP4 levels have been observed in humans with obesity and/or type 2 diabetes [3].

Nevertheless, the correlations between RBP4 and insulin resistance from several cross-sectional studies with small sample sizes have yielded inconsistent results [6–15]. Furthermore, inherent to the limitation of the methodology, the temporal relation between RBP4 and the risk of diabetes cannot be established from cross-sectional studies. To our best knowledge, only two cohort studies have examined the association between RBP4 and type 2 diabetes risk in general populations: the Atherosclerosis Risk in Communities (ARIC) study reported a positive association only in women, but not in men or total study population [16]; while a cohort study among a Chinese population observed a positive association in total study participants but did not examine potential heterogeneity by sex [17]. Therefore, it remains unclear whether RBP4 will be a useful risk marker for type 2 diabetes in both men and women.

Therefore, to examine sex-specific association between plasma RBP4 levels and type 2 diabetes in a prospective manner, we first conducted a case-control study nested within the Singapore Chinese Health Study cohort, with comprehensive adjustment for potential confounders and subgroup analyses. We then obtained sex-specific results from the previous two cohort studies, and combined them with our results in a meta-analysis.

## Methods

### Study population

The detailed design of the Singapore Chinese Health Study has been described previously [18]. Briefly, baseline recruitment was conducted between 1993 and 1998, and a total of 63,257 Chinese adults aged 45–74 years old were recruited. At follow-up I (1999–2004), 52,322 participants were re-contacted successfully and among them, 32,535 subjects donated blood for our research. At follow-up II (2006–2010), 39,528 participants were re-contacted successfully, and among them, 25,477 were blood donors at follow-up I (78.3% of this sub-cohort). The study protocol was approved by the Institutional Review Boards at the National University of Singapore and the University of Pittsburg, and all participants gave written informed consent at the baseline interview.

### Assessment of covariates and outcome

During the baseline and both follow-up interviews, information regarding age, self-reported height and weight, smoking status, alcohol intake, menopausal status (for women), and history of hypertension, cardiovascular disease (CVD), stroke and cancer was collected via in-person interviews using structured questionnaires. Body mass index (BMI) was calculated using body weight (in kilograms) divided by the square of height (in meters). In addition, baseline interviews also included information on sex, dialect group, highest educational level, a detailed dietary assessment using a validated semi-quantitative food frequency questionnaire and time spent per week in various types of physical activities.

A history of physician-diagnosed type 2 diabetes was solicited at all interviews by asking the participants the question “Have you been told by a doctor that you have diabetes?”, and participants who gave positive answers were also asked for the age at first diagnosis. In a separate validation study, this self-report of type 2 diabetes in this cohort has shown to be valid and robust [19].

### Establishment of the nested case-control study

For the current analysis, we established a nested case-control study within this cohort among those who donated blood samples at follow-up I and also participated in all interviews. A total of 571 participants reported that they had no diabetes at follow-up I but reported to have diabetes at follow-up II, and were defined as incident type 2 diabetes cases. Controls were selected among those who reported that they had no diabetes at both follow-up interviews, and also with HbA_1c_ levels less than 6.0% measured in the blood donated at follow-up I. Cases and controls were matched for age (±3 years), sex, dialect group (Cantonese, Hokkien), and date of blood collection (±6 months) on a 1:1 ratio. The flowchart of the study design is shown in Additional file 1: Figure S1.

### Laboratory measurements

A 20-mL morning blood was collected from each participant that agreed to donate blood. The blood was then put on ice and transported to the laboratory immediately. Subsequently the blood was separated into different components (e.g. serum, plasma, buffy coat, and red blood cells) and kept at − 80 °C freezers for long-term storage. For the current study, we further measured the levels of various biomarkers using the stored blood. Plasma concentrations of RBP4 were measured via Sandwich ELISA (Bio-Rad Laboratories, Hercules, CA), of which the within-assay and between-assay coefficients of variation were 5.7–8.1% and 5.8–8.6%, respectively. In addition, the measurements of other biomarkers including adiponectin, ferritin, total cholesterol (TC), HDL-cholesterol (HDL-C), triglycerides (TG), high-sensitivity C-reactive protein (hs-CRP), alanine aminotransferase (ALT), random insulin and hemoglobin A1c (HbA1c) levels were described in details in previous studies [20–23].

### Statistical analysis

Characteristics were presented for men and women separately because of the different distributions by sex. Study subjects were divided according to sex-specific quartiles of RBP4 levels among control participants, and the lowest quartile was used as the reference group. We used conditional logistic regression models to compute the odds ratio (OR) and 95% confidence interval (CI) between RBP4 and risk of type 2 diabetes. In model 1, we adjusted for established risk factors for type 2 diabetes such as age (years, continuous), education levels (none, primary, secondary or higher), smoking status (never, past, and current smoker), alcohol consumption (never/monthly, weekly or daily), weekly duration of moderate-to-vigorous physical activity (< 0.5, 0.5–3.9, and ≥ 4.0 h/week), history of hypertension (yes, no), fasting status at blood donation (yes, no) and BMI (kg/m^2^, continuous). In addition, since previous studies have suggested that RBP4 may be implicated in the pathogenesis of type 2 diabetes via etiologic pathways of insulin resistance [3], inflammation [24], and failure of intracellular lipid homeostasis [25], we additionally adjusted for surrogate biomarkers of the abovementioned pathways, including hs-CRP (mg/L), adiponectin (μg/mL), TG (mmol/L), HDL-C (mmol/L) and ALT (IU/L) in model 2 to examine whether the association between RBP4 and type 2 diabetes would be independent of or mediated through these etiological pathways. Subsequently we conducted the abovementioned analysis stratified by sex, and we additionally adjusted for menopausal status in women. In addition, we used restricted cubic spline regression to examine for a potential non-linear relation between RBP4 and risk of type 2 diabetes, with 4 knots at 5, 35, 65 and 95% percentiles of the original value of RBP4 concentrations. Moreover, we tested potential effect modification by age (< 60 versus ≥60 years), BMI (< 23 versus ≥23 kg/m^2^), alcohol consumption (never versus weekly or daily), physical activity (< 0.5 versus ≥0.5 h/week), fasting status at blood donation (yes versus no), plasma levels of ferritin, hs-CRP, adiponectin, ALT, TG, HDL-C (above versus below median levels of each biomarker) and HbA1c levels (< 6.5% versus ≥6.5%) by adding an interaction term (each binary variable × RBP4 levels in quartile categories) to the regression models in men and women separately. Since previous studies have suggested that circulating RBP4 concentration might reflect insulin resistance-associated iron overload reflected by ferritin levels [26], we also examined for potential interaction between ferritin and RBP4 in association with risk of type 2 diabetes (above versus below median level of ferritin). In the above stratified analyses, unconditional logistic regression models were applied, with additional adjustment for sex and dialect group (Hokkien, Cantonese).

We pooled our multivariable-adjusted risk estimates with those from prior cohort studies of RBP4 and type 2 diabetes using a meta-analysis approach. As we only included prospective cohort studies that were conducted in general populations, we excluded one study conducted among Asian Indian men with prediabetes [27]. We used the DerSimonian-Laird random-effect models to pool the relative risks (RRs), represented either by ORs or hazard ratios comparing the highest versus lowest category of RBP4 levels (tertiles or quartiles), and the corresponding 95% CIs [28]; and used fixed-effect model in sensitivity analyses. We used Cochran χ^2^ and *I*^*2*^ Statistic to evaluate the heterogeneity among studies [29]. To further examine the influence of sex on the observed association, we contacted the authors of the previous publications and obtained the sex-specific results. We performed all analyses with Stata software, version 14.0 (Stata Corp, College Station, Texas), and considered two-sided *P* values < 0.05 to be statistically significant.

## Results

Of all the subjects that were re-contacted successfully in the follow-up I interview, 32,535 subjects (approximately 62%) consented to donating blood for research. The study participants who provided blood samples were younger than those who did not (mean ± SD: 60.9 ± 7.7 versus 62.4 ± 8.2 years of age), more educated (33.6% versus 25.1% having secondary or higher education), more likely to be men (45.5% versus 39.1%), and had slightly higher prevalence of smoking (32% versus 30% ever smokers) and regular alcohol consumption (18.3% versus 14.8% weekly or daily consumers of alcohol). However, the prevalence of type 2 diabetes were similar among participants who donated blood and who did not (13.8% versus 13.7%).

The sex-specific characteristics of cases with type 2 diabetes and matched controls in this study are presented in Table 1. Compared to matched controls, cases had higher BMI and were more likely to have hypertension in both men and women. In addition, majority of women (84.7%) were postmenopausal at the time of blood donation. No significant differences were found for levels of education, physical activity and alcohol consumption between cases and controls, and controls were more likely to be never smokers among women. For blood biomarkers, compared to controls, cases had higher concentrations of ferritin, hs-CRP, TG, ALT, HbA1c, insulin and glucose, but lower levels of adiponectin and HDL-C in both men and women. Plasma RBP4 levels were significantly higher in men than women, and the median values were 30 (interquartile range [IQR]: 24–35) μg/mL in men, and 25 (IRQ: 21–31) μg/mL in women. In comparison by status of incident diabetes, among men, the median (IQR) concentrations of RBP4 were 30 (24–36) μg/mL among cases and 30 (25–34) μg/mL among controls in men (*P* = 0.93). Correspondingly, among women, the levels were 27 (22–33) μg/mL among cases and 24 (21–30) μg/mL among controls (*P* < 0.001). Furthermore, the median (IQR) of RBP4 was higher among post-menopausal women (median 26; IQR 22–32 μg/mL) than pre-menopausal women (median 22; IQR 18–30 μg/mL). Overall, plasma RBP4 levels were positively correlated with TC, TG, ALT and ferritin, and negatively correlated with adiponectin, regardless of case-control status (Additional file 1: Table S1) or sex (data not shown).

**Table 1:** Characteristics of diabetes cases and matched controls in men and women^*^

	Men			Women		
	Cases (*n* = 236)	Controls (*n* = 236)	*P*-value^†^	Cases (*n* = 335)	Controls (*n* = 335)	*P*-value^†^
Age at blood taken, years	60.3 ± 6.23	60.3 ± 6.32	–	59.2 ± 6.03	59.3 ± 6.11	–
Dialect (%)			–			–
Cantonese	107 (45.3)	107 (45.3)		180 (53.0)	180 (53.0)	
Hokkien	129 (54.7)	129 (54.7)		155 (46.3)	155 (46.3)	
Body mass index, kg/m^2^	24.6 ± 3.60	22.9 ± 3.26	< 0.001	24.9 ± 3.64	22.7 ± 3.29	< 0.001
Menopausal status			–			0.75
Premenopausal	–	–		49 (14.6)	52 (15.5)	
Postmenopausal	–	–		286 (85.4)	283 (84.5)	
Level of education (%)			0.21			0.80
No formal education	12 (5.08)	11 (4.66)		92 (27.5)	88 (26.3)	
Primary school	119 (50.4)	101 (42.8)		136 (40.6)	132 (39.4)	
Secondary and above	105 (44.5)	124 (52.5)		107 (31.9)	115 (34.3)	
History of hypertension (%)	108 (45.8)	62 (26.3)	< 0.001	157 (46.9)	86 (25.7)	< 0.001
Cigarette smoking (%)			0.13			0.04
Never smokers	100 (42.4)	101 (42.8)		310 (92.5)	324 (96.7)	
Former smoker	52 (22.0)	68 (28.8)		11 (3.3)	3 (0.9)	
Current smokers	84 (35.6)	67 (28.4)		14 (4.2)	8 (2.4)	
Weekly moderate activity (%)			0.29			0.34
< 0.5 h/week	182 (77.1)	184 (78.0)		274 (81.8)	270 (80.6)	
0.5–3.9 h/week	39 (16.5)	30 (12.7)		43 (12.8)	38 (11.3)	
≥ 4 h/week	15 (6.36)	22 (9.32)		18 (5.4)	27 (8.1)	
Alcohol intake (%)			0.45			0.28
Abstainers	183 (77.5)	193 (81.8)		315 (94.0)	304 (90.8)	
Weekly drinkers	38 (16.1)	33 (14.0)		17 (5.1)	26 (7.8)	
Daily drinkers	15 (6.36)	10 (4.24)		3 (0.9)	5 (1.5)	
Fasting status (yes, %)	71 (30.1)	68 (28.8)	0.76	107 (31.9)	88 (26.3)	0.11
RBP4, μg/mL	30 (24–36)	30 (25–34)	0.93	27 (22–33)	24 (21–30)	0.001
Ferritin, μg/L	236 (151–337)	179 (115–251)	< 0.001	150 (85–230)	104 (63–160)	< 0.001
ALT, IU/L	30 (22–40)	22 (17–29)	< 0.001	25 (19–34)	19 (14–25)	< 0.001
TC, mmol/L	5.05 ± 0.82	5.12 ± 0.97	0.37	5.43 ± 0.91	5.31 ± 0.86	0.06
HDL-C, mmol/L	0.99 ± 0.19	1.10 ± 0.28	< 0.001	1.15 ± 0.24	1.32 ± 0.32	< 0.001
TG, mmol/L	2.2 (1.5–3.2)	1.7 (1.2–2.4)	< 0.001	2.1 (1.5–2.8)	1.5 (1.0–2.0)	< 0.001
Adiponectin, μg/mL	6.31 ± 2.40	7.67 ± 2.75	< 0.001	7.48 ± 2.79	10.1 ± 4.05	< 0.001
Hs-CRP, mg/L	1.5 (0.8–2.9)	1.1 (0.4–2.1)	0.001	2.3 (1.2–4.2)	1.3 (0.7–2.5)	< 0.001
Random insulin, mIU/L	15.3 (7.6–37.8)	8.5 (4.4–21.6)	< 0.001	14.4 (8.2–33.8)	9.0 (4.5–21.7)	< 0.001
Random glucose, mmol/L	6.0 (4.8–8.8)	4.5 (4.0–5.3)	< 0.001	6.1 (4.8–9.0)	4.6 (4.1–5.4)	< 0.001
HbA_1c_, %	6.4 (5.9–7.0)	5.6 (5.4–5.8)	< 0.001	6.5 (6.0–7.2)	5.6 (5.4–5.8)	< 0.001
HbA_1c_, mmol/mol	46 (41–53)	38 (36–40)	< 0.001	48 (42–55)	38 (36–40)	< 0.001

^*^Data are expressed as mean ± standard deviation for continuous variables (normally distributed) and median (interquartile range) for continuous variables (skewed distributed), and *n* (percentage) for categorical variables. Cases and controls are matched on age at blood taken (±3 years), gender, dialect (Cantonese, Hokkien), and date of blood collection (±6 months).

^†^*P* values based on the chi-square test for categorical variables, student’s t-test for normally-distributed continuous variables and Mann-Whitney test for skewed continuous variable.

Abbreviations: *ALT* alanine aminotransferase, *HbA*_*1c*_ hemoglobin A1c, *HDL-C* HDL cholesterol, *hs-CRP* high-sensitivity C-reactive protein, *RBP4* retinol binding protein 4, *TC* total cholesterol, *TG* triglycerides

The association between RBP4 and type 2 diabetes risk is presented in Table 2. In the whole study population, after adjustment for age, sex, lifestyle factors, fasting status and BMI, raised levels of plasma RBP4 were significantly associated with higher risk of type 2 diabetes; the OR (95% CI) comparing the last quartile against the first quartile was 1.69 (1.09–2.62; *P*-trend =0.004). After additional adjustment for TG, HDL-C, hs-CRP and ALT, although the association was attenuated and became non-significant in the whole population in model 2 (OR 1.23; 95% CI 0.73–2.07; *P*-trend =0.14), the positive association remained present in women but not in men, and this interaction with sex was statistically significant (*P*-interaction =0.032). Comparing the extreme quartiles of RBP4 levels in model 2, the OR (95% CI) was 2.29 (1.05–5.00; *P*-trend =0.018) in women, and 0.63 (0.27–1.45; *P*-trend =0.65) in men. In addition, the cubic spline regression analysis using variables from the model 2 suggested that the relationship between RBP4 and type 2 diabetes risk was linear and the *P*-nonlinearity value was 0.40 in men and 0.18 in women (Additional file 1: Figure S2). Given the linear association, we also estimated the risk of type 2 diabetes associated with every 1-log μg/mL increment in RBP4 levels, and the OR (95% CI) in model 2 was 1.38 (0.71–2.65) in the whole study population, 0.63 (0.21–1.84) in men and 2.78 (1.09–7.14) in women. Further adjusting for menopausal status in women made little impact on the RBP4-diabetes association in women (OR per log RBP4 was 2.84; 95% CI 1.09–7.42).

**Table 2:** Odds ratios (95% confidence intervals) of type 2 diabetes associated with different levels of RBP4

Variables	Quartiles of RBP4				*P* for trend^*^	Per 1 log increment
	Q1	Q2	Q3	Q4		
Total						
Median (range)	19 (11–22)	24 (22–26)	30 (26–33)	38 (33–77)		
Cases/controls	125/143	121/143	149/143	176/143		
Model 1^†^	1.00	0.92 (0.60–1.41)	1.34 (0.89–2.02)	1.69 (1.09–2.62)	0.004	2.09 (1.20–3.64)
Model 2^‡^	1.00	0.70 (0.42–1.18)	1.12 (0.68–1.84)	1.23 (0.73–2.07)	0.14	1.38 (0.71–2.65)
Men						
Median (range)	21 (14–25)	27 (25–30)	32 (30–34)	40 (35–76)		
Cases/controls	67/59	53/59	48/59	68/59		
Model 1^†^	1.00	0.55 (0.27–1.11)	0.57 (0.29–1.12)	0.94 (0.47–1.86)	0.81	1.04 (0.42–2.54)
Model 2^‡^	1.00	0.37 (0.16–0.86)	0.53 (0.24–1.16)	0.63 (0.27–1.45)^§^	0.65	0.63 (0.21–1.84)
Women						
Median (range)	18 (11–21)	23 (21–24)	27 (24–30)	36 (30–77)		
Cases/controls	58/84	68/84	101/84	108/83		
Model 1^†^	1.00	1.41 (0.79–2.51)	2.55 (1.46–4.48)	2.59 (1.41–4.75)	0.001	3.46 (1.66–7.20)
Model 2^‡^	1.00	1.17 (0.55–2.50)	2.43 (1.15–5.12)	2.29 (1.05–5.00)^§^	0.018	2.78 (1.09–7.14)

^*^Linear trend was tested by using the median level of each quartile of RBP4

^†^Model 1: adjusted for age at blood taken (continuous), smoking status (never, past, and current smoker), alcohol intake (never, weekly or daily), weekly moderate-to-vigorous activity levels (<0.5, 0.5-3.9, and ≥4.0 hours/week), education level (primary school and below, secondary or above), history of hypertension (yes, no), fasting status (yes, no), and body mass index (continuous);

^‡^Model 2: Model 1 plus hs-CRP (mg/L), TG (mmol/L), HDL-C (mmol/L), and ALT (IU/L) (all in quartiles).

^§^*P*-interaction, men vs. women =0.032 in Model 2.

Abbreviations: *ALT* alanine aminotransferase, *HDL-C* high-density lipoprotein cholesterol, *hs-CRP* high-sensitivity C-reactive protein, *RBP4* retinol binding protein 4, *TG* triglycerides

We then examined if the association between RBP4 and type 2 diabetes risk was different among participants with different characteristics in men and women separately (Table 3 & Additional file 1: Table S2). In women, the RBP4-diabetes association was significantly stronger among subjects with lower ALT levels (below median of 21.5 IU/L) than those with higher ALT concentrations (*P*-interaction =0.004), and it was stronger among individuals with low ferritin levels (below median 150 μg/mL) than those with higher ferritin concentrations (*P*-interaction =0.03; Table 3). The differences in association by BMI (*P*-interaction =0.14) or ferritin levels (*P*-interaction =0.16) among women did not reach statistical significance. In men, all variables that we had examined did not modify the risk association between RBP4 and type 2 diabetes, and the RBP4-diabetes association stayed largely null and non-significant in all subgroups (Additional file 1: Table S2).

**Table 3:** Odds ratios (95% confidence intervals) of type 2 diabetes associated with different levels of RBP4 stratified by various factors in women

Variables	Quartiles of RBP4					*P-*trend	*P*-interaction
	n	Q1	Q2	Q3	Q4		
Age (year)^*^							
< 60	390	1.00	1.19 (0.60–2.33)	1.29 (0.69–2.44)	2.06 (1.07–3.96)	0.03	0.85
≥ 60	280	1.00	1.03 (0.45–2.34)	2.75 (1.22–6.18)	1.61 (0.76–3.44)	0.11	
BMI^*^, kg/m^2^							
< 23	293	1.00	1.69 (0.76–3.75)	2.06 (0.99–4.29)	2.71 (1.25–5.88)	0.01	0.14
≥ 23	377	1.00	0.85 (0.43–1.66)	1.58 (0.81–3.10)	1.25 (0.66–2.37)	0.23	
Alcohol consumption^*^							
Never	619	1.00	1.09 (0.64–1.85)	1.80 (1.09–2.98)	1.85 (1.12–3.06)	0.004	0.49
Weekly or daily	51	1.00	6.37 (0.49–82.8)	5.41 (0.44–66.5)	1.51 (0.10–23.6)	0.91	
Physical activity^*^							
< 0.5 h/week	544	1.00	1.25 (0.71–2.22)	1.90 (1.11–3.27)	1.83 (1.08–3.11)	0.01	0.95
≥ 0.5 h/week	126	1.00	0.63 (0.18–2.25)	1.39 (0.42–4.60)	1.47 (0.43–5.03)	0.24	
Menopausal status^*^							
Premenopausal	101	1.00	1.67 (0.39–7.18)	1.23 (0.32–4.70)	2.66 (0.70–10.2)	0.20	0.76
Postmenopausal	569	1.00	1.12 (0.63–1.96)	2.01 (1.17–3.45)	1.80 (1.06–3.08)	0.01	
Fasting status^*^							
Fasting	195	1.00	1.52 (0.58–3.99)	1.15 (0.46–2.91)	1.98 (0.82–4.77)	0.18	0.81
Non-fasting	475	1.00	1.00 (0.54–1.86)	2.15 (1.20–3.83)	1.72 (0.95–3.10)	0.02	
Ferritin^*^, μg/mL							
< 150	275	1.00	1.28 (0.66–2.48)	1.73 (0.91–3.27)	2.03 (1.06–3.88)	0.02	0.03
≥ 150	278	1.00	1.93 (0.37–10.1)	1.60 (0.39–6.62)	0.67 (0.19–2.39)	0.19	
Hs-CRP^*^, mg/L							
< 1.7	329	1.00	1.22 (0.52–2.83)	2.64 (1.23–5.66)	2.60 (1.19–5.66)	0.004	0.16
≥ 1.7	341	1.00	1.29 (0.63–2.62)	1.49 (0.76–2.95)	1.49 (0.77–2.90)	0.25	
Adiponectin^*^, μg/mL							
< 8.1	326	1.00	1.61 (0.72–3.61)	2.09 (0.97–4.50)	1.64 (0.80–3.37)	0.26	0.39
≥ 8.1	344	1.00	0.92 (0.44–1.95)	1.83 (0.90–3.70)	1.51 (0.72–3.16)	0.10	
ALT^*^, IU/L							
< 21.5	335	1.00	1.06 (0.49–2.30)	3.01 (1.49–6.11)	2.69 (1.31–5.53)	0.001	0.004
≥ 21.5	335	1.00	1.25 (0.56–2.83)	0.99 (0.47–2.07)	0.99 (0.48–2.05)	0.74	
TG^*^, mmol/L							
< 1.72	335	1.00	0.95 (0.47–1.92)	1.11 (0.56–2.19)	1.26 (0.62–2.58)	0.45	0.79
≥ 1.72	335	1.00	0.90 (0.38–2.13)	1.84 (0.81–4.16)	1.27 (0.57–2.81)	0.34	
HDL-C^*^, mmol/L							
< 1.18	329	1.00	1.62 (0.77–3.42)	2.68 (1.25–5.72)	1.59 (0.80–3.18)	0.22	0.54
≥ 1.18	341	1.00	0.77 (0.36–1.66)	1.56 (0.78–3.10)	1.79 (0.87–3.66)	0.02	
HbA1c^†^, %							
< 6.5	328	1.00	2.20 (0.85–5.65)	3.47 (1.40–8.60)	5.13 (1.91–13.8)	0.001	0.19
≥ 6.5	342	1.00	1.03 (0.43–2.46)	2.09 (0.93–4.71)	1.61 (0.68–3.80)	0.11	

^*^Odds ratio was estimated using unconditional logistic regression model adjusted for age at blood taken (continuous), dialect group (Cantonese, Hokkien), smoking status (never, past, and current smoker), alcohol intake (never, weekly or daily), weekly moderate-to-vigorous activity levels (<0.5, 0.5-3.9, and ≥4.0 hours/week), education level (primary school and below, secondary or above), history of hypertension (yes, no), fasting status (yes, no), body mass index (continuous), and menopausal status (premenopausal, postmenopausal).

^†^Odds ratio was estimated using conditional logistic regression model adjusted for abovementioned variables except for dialect group (Cantonese, Hokkien).

Abbreviations: *ALT* alanine aminotransferase, *BMI* body mass index, *HbA1c* Hemoglobin A1c, *HDL-C* high-density lipoprotein cholesterol, *hs-CRP* high-sensitivity C-reactive protein, *RBP4* retinol binding protein 4, *TG* triglycerides

**Fig. 1:**
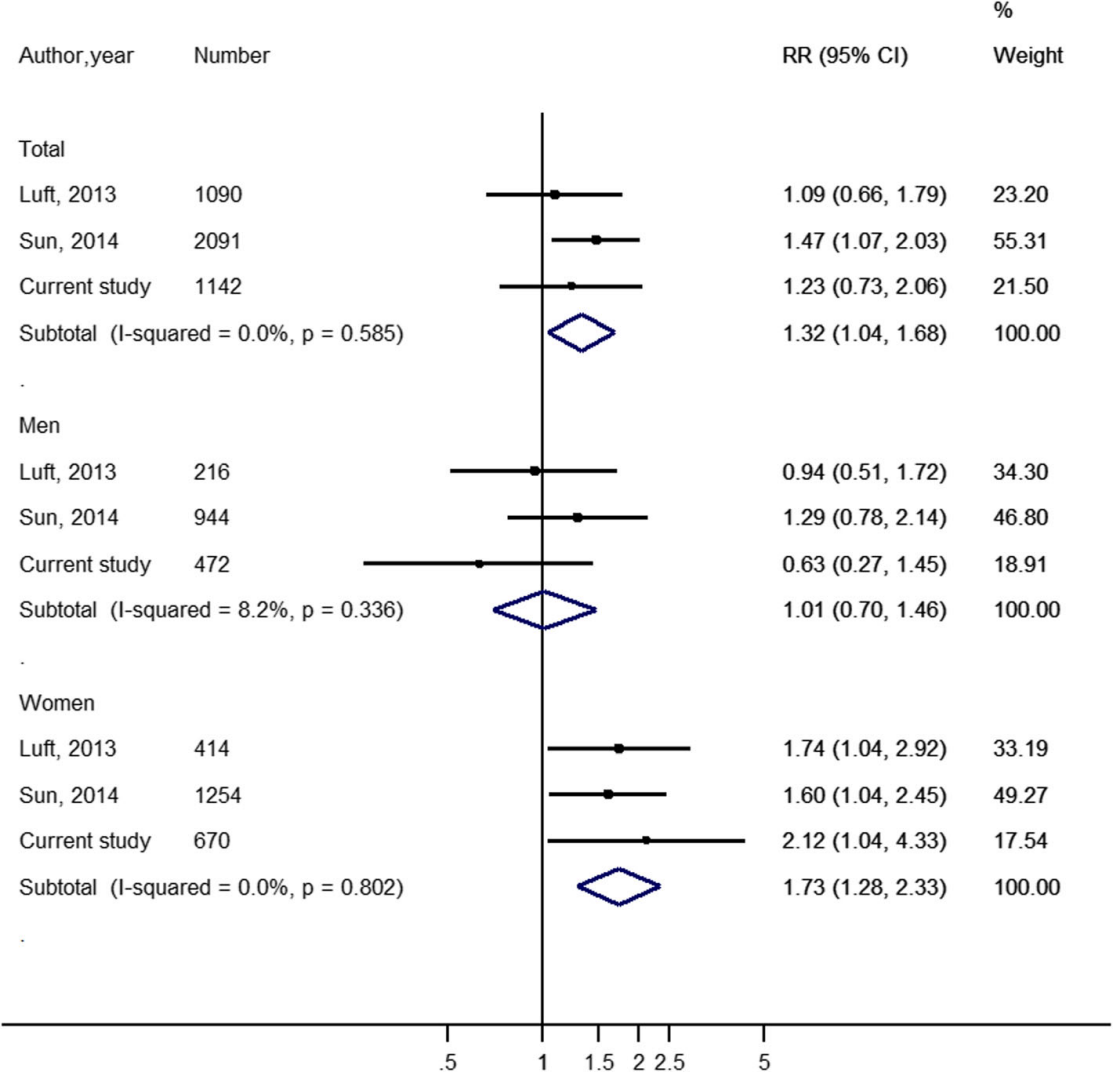
Adjusted relative risk of retinol binding protein 4 levels with risk of type 2 diabetes in meta-analysis. The relative risk (RR) of incident diabetes was obtained from each study using a random-effects model. The data markers indicate the adjusted RRs comparing highest versus lowest category of RBP4 levels. The size of the data markers indicates the weight of the study, which is the inverse variance of the effect estimate. The diamond data markers indicate the pooled RRs

When we pooled our results with those of two previous prospective studies [16, 17], we observed consistent results across all three studies. The pooled RR (95% CI) using random-effects model was 1.32 (1.04–1.68; *I*^*2*^ = 0%; *P* for heterogeneity =0.59) when comparing highest versus lowest RBP4 levels in the total population, and it was 1.01 (0.70–1.46; *I*^*2*^ = 8.2%; *P* for heterogeneity =0.34) in men and 1.73 (1.28–2.33; *I*^*2*^ = 0%; *P* for heterogeneity =0.80) in women (Fig. 1). Results were similar when the fixed-effects model was used (data not shown). A detailed description of the characteristics of these studies is listed in Additional file 1: Table S3.

## Discussion

In this Chinese population living in Singapore, we found a strong dose-dependent association between higher plasma RBP4 levels and increased risk of type 2 diabetes in women but not in men after adjusting for other established risk factors of type 2 diabetes, including lipids (TG, HDL-C), inflammation marker (hs-CRP), liver enzyme (ALT) and adiponectin. This sex-specific association concurred with the findings from the meta-analysis involving our study and two prior prospective studies.

To our best knowledge, hitherto, only two cohort studies have examined the prospective association between RBP4 and type 2 diabetes risk. In agreement with our null finding in the total population, a 9-year prospective cohort study among 1090 African American and Caucasian men and women in the US, with 543 incident cases of type 2 diabetes, has also reported a HR of 1.09 (95% CI 0.66–1.79) when comparing the extreme tertiles of RBP4 concentration [16]. In contrast, another 6-year follow-up study among 2091 Chinese men and women, with 507 cases of type 2 diabetes, has shown a positive RBP4-diabetes association in the overall population, and the HR comparing the highest versus lowest quintile of RBP4 concentration was 1.47 (95% CI 1.07–2.03) [17]. However, despite the inconsistent results in the total population, our meta-analysis has shown a consistent sex-specific association in our study and these two studies, where the positive association was only observed in women but not in men. Nevertheless, the prospective study that was excluded from the current meta-analysis has reported a significant association between RBP4 and type 2 diabetes risk among 147 Asian Indian men with prediabetes (OR per SD: 1.65; 95% CI 1.03–2.66) [27]. Higher RBP4 levels have been reported in human subjects with impaired glucose tolerance compared to those with normal glucose tolerance [30]. However, since no other study has examined the impact of impaired glucose tolerance on the association between RBP4 and type 2 diabetes risk in men with prediabetes, further studies are warranted.

Mechanistic studies have shown that RBP4 may be involved in several etiologic pathways leading to type 2 diabetes development, such as dysregulation of insulin resistance and insulin secretion, inflammation, and failure of intracellular lipid homeostasis. RBP4 has shown to decrease insulin sensitivity by inducing liver expression of phosphoenolpyruvate kinase [3] or stimulating inflammation in adipocytes [24]. In addition to inducing insulin resistance, RBP4 may also have impact on insulin secretion from beta-cells, as genetic studies have shown that SNP risk alleles rs3758539 (A) and rs34571439 (G) in the *RBP4* gene were associated with reduced insulin secretion [31, 32]. Therefore, in this study, we have examined whether markers of insulin resistance, inflammation, and non-alcoholic fatty liver disease could mediate the association between RBP4 and type 2 diabetes (model 2 of Table 2), our study and other previous studies [16, 17] showed that the association between RBP4 and diabetes remained significant in women after adjusting for markers of insulin resistance (HOMA-IR, random insulin levels), inflammation (hs-CRP), and liver enzymes (Gamma-glutamyltransferase, ALT). This suggests that the association between RBP4 and type 2 diabetes was independent of those pathways in women.

Although the underlying mechanisms for the observed sex-heterogeneity are not fully understood yet, estrogen may have a regulatory role in RBP4 levels and this may explain the interaction between RBP4 levels and sex in influencing type 2 diabetes risk. Animal studies have suggested that estrogen could regulate RBP4 expression through retinoic acid metabolism [33, 34]. In humans, a cross-sectional study in Chinese women has found a negative correlation between estrogen levels and RBP4 levels [35]. Consistently, our study and another study among Chinese women have both reported higher RBP4 levels among post-menopausal women compared to pre- menopausal women [36]. Nevertheless, the RBP4-diabetes association was not different by menopausal status in our study. Since we did not have information on estrogen level, whether estrogen plays an important role in the association between RBP4 and type 2 diabetes risk remains to be explored, and more mechanistic studies are needed to elucidate the reason for the difference in results between men and women.

Furthermore, we have observed a stronger RBP4-diabetes association among women with low levels of ALT or ferritin levels (< median values) in the current study. Although the mechanism is not fully understood yet, our previous studies in the same population have shown that elevated plasma levels of ALT and ferritin are both independent risk factors for type 2 diabetes [21, 23]. Therefore, one possible explanation for the observed heterogeneity between subgroups may be difference in the dominant pathogenesis pathway driving type 2 diabetes development. Among low-risk participants who have lower ALT or ferritin levels, increased RBP4 may play a major role in type 2 diabetes development, while in their respective counterparts who are at higher risk of type 2 diabetes due to higher ALT or ferritin levels, other risk factors such as inflammation may play a more prominent role. We have previously observed in this cohort that elevated inflammatory status (indicated by higher levels of hs-CRP) was significantly associated with increased risk of type 2 diabetes in the presence of raised ferritin levels [21]. However, given that multiple statistical tests for interaction were conducted, our observed interaction may also be due to chance and should be interpreted with caution.

Some limitations merit consideration in the current study. First, cases and controls were chosen from 57% of the cohort participants who donated blood samples, and this may have introduced selection bias. However, despite differences in the distributions of some demographic characteristics between participants who donated blood and those who did not donate, they were largely comparable for risk factors of diabetes. Moreover, the prevalence of type 2 diabetes was similar between these two groups (13.8% versus 13.7%). Since we measured RBP4 only once, some random measurement errors may exist, which could lead to non-differential misclassification of type 2 diabetes status and an underestimation of the true association. In addition, residual confounding may exist in the use of self-reported height, weight and history of hypertension, and information on family history of type 2 diabetes and pharmacological therapy that may affect glucose and RBP4 levels was not collected. Although 70% of blood samples were taken at non-fasting state, we have compared RBP4 levels at blood donation by fasting status and performed stratified analysis, and found that neither RBP4 concentration nor the association with type 2 diabetes risk differed between the two groups, indicating that fasting status did not influence the associations in the present study. Finally, incident diabetes was self-reported and undiagnosed diabetes may exist at blood taking. However, we have conducted sensitivity analysis restricted to cases with HbA1c < 6.5% at blood taking and their respective controls, and observed similar association between RBP4 and type 2 diabetes, thus suggesting that undiagnosed diabetes was unlikely to impact the association between RBP4 and type 2 diabetes.

## Conclusions

We observed a strong, dose-dependent association between plasma RBP4 levels and increased risk of incident type 2 diabetes in Chinese women but not men in our study, and confirmed this finding in a meta-analysis that further included two previous prospective studies. Further research is needed to validate the findings and to investigate the biological mechanisms for our observation. Recent studies have shown that pharmacological therapy, weight loss and exercise could decrease RBP4 levels and improve insulin sensitivity [3, 37, 38]. Therefore, more studies with sufficient sample size in men and women separately are needed to examine the feasibility of targeting RBP4 through these pharmacological and lifestyle interventions to reduce the risk of type 2 diabetes in high-risk populations.

## Additional file

Additional file 1: **Table S1.** The pair-wise Pearson correlation coefficients between RBP4 and age, body mass index, and plasma levels of blood biomarkers. **Table S2.** Odds ratios (95% confidence intervals) of type 2 diabetes by stratified analysis in men **Table S3.** Prospective studies of RBP4 and incident type 2 diabetes. **Figure S1.** Flowchart of the Singapore Chinese Health Study. **Figure S2.** Spline analysis of the association between plasma levels of RBP4 and incident type 2 diabetes in women (A) and men (B). (DOCX 167 kb)

## Abbreviations

ALT: Alanine transaminase
CI: Confidence interval
HbA1c: Hemoglobin A1c
HDL-C: High-density lipoprotein cholesterol;
HR: Hazard ratio
Hs-CRP: High-sensitivity C-reactive protein
OR: Odds ratio
RBP4: Retinol binding protein 4
RR: Relative risk
SD: Standard deviation
TC: Total cholesterol
TG: Triglycerides
