# Supplementary material for: Retinol binding protein 4 and risk of type 2 diabetes in Singapore Chinese men and women: a nested case-control study
Source: Nutr Metab (Lond). 2019 Jan 10;16:3. doi: 10.1186/s12986-018-0329-0 (PMC6329092; doi:10.1186/s12986-018-0329-0)
Supplement: Supplementary file 1 — Table S1. The pair-wise Pearson correlation coefficients between RBP4 and age, body mass index, and plasma levels of blood biomarkers. Table S2. Odds ratios (95% confidence intervals) of type 2 diabetes by stratified analysis in men Table S3. Prospective studies of RBP4 and incident type 2 diabetes. Figure S1. Flowchart of the Singapore Chinese Health Study. Figure S2. Spline analysis of the association between plasma levels of RBP4 and incident type 2 diabetes in women (A) and men (B). (DOCX 167 kb) [file 12986_2018_329_MOESM1_ESM.docx]

**Retinol binding protein 4 and risk of incident type 2 diabetes in Singapore Chinese men and women: a nested case-control study**

Wang Y, Sun L, Lin X, Yuan JM, Koh WP, Pan A

**Online Supplemental Material**

The following materials are included in the Online Supplemental Material.

1. Supplemental Table S1
2. Supplemental Table S2
3. Supplemental Table S3
4. Supplemental Figure S1
5. Supplemental Figure S2

**Supplemental Table S1.** The pair-wise Pearson correlation coefficients between RBP4 and age, body mass index, and plasma levels of blood biomarkers

**Supplemental Table S2.** Odds ratios (95% confidence intervals) of type 2 diabetes by stratified analysis in men

**Supplemental Table S3.** Prospective studies of RBP4 and incident type 2 diabetes

**Supplemental Figure S1.** Flowchart of the Singapore Chinese Health Study

**Supplemental Figure S2.** Spline analysis of the association between plasma levels of RBP4 and incident type 2 diabetes in women (A) and men (B)

**Supplemental Table S1.** The pair-wise Pearson correlation coefficients between RBP4 and age, body mass index, and plasma levels of blood biomarkers

| Marker | Controls | Cases | Total |
| --- | --- | --- | --- |
| Age | 0.08^a^ | 0.07 | 0.08^b^ |
| BMI | 0.01 | -0.03 | 0.01 |
| Ferritin | 0.26^b^ | 0.15^b^ | 0.21^b^ |
| ALT | 0.28^b^ | 0.04 | 0.18^b^ |
| TC | 0.15^b^ | 0.17^b^ | 0.17^b^ |
| HDL-C | -0.10^a^ | -0.01 | -0.08^b^ |
| TG | 0.29^b^ | 0.27^b^ | 0.29^b^ |
| Hs-CRP | 0.01 | -0.09^a^ | -0.03 |
| Adiponectin | -0.25^b^ | -0.11^a^ | -0.20^b^ |
| Random insulin | 0.05 | -0.01 | 0.04 |
| Random glucose | -0.05 | -0.07 | -0.02 |

^a^Correlation coefficients are statistically significant at *P* <0.05;

^b^Correlation coefficients are statistically significant at *P* <0.01;

**Abbreviations:** ALT, alanine aminotransferase; BMI, body mass index; HDL-C, high-density lipoprotein cholesterol; hs-CRP, high-sensitivity C-reactive protein; RBP4, retinol binding protein 4; TC, total cholesterol; TG, triglycerides;.

**Supplemental Table S2.** Odds ratios (95% confidence intervals) of type 2 diabetes associated with different levels of RBP4 stratified by various factors in men^a^

| Variables | Quartiles of RBP4 | | | | | *P-*trend | *P*-interaction |
| --- | --- | --- | --- | --- | --- | --- | --- |
|  | n | Q1 | Q2 | Q3 | Q4 |  |  |
| Age (year)^a^ |  |  |  |  |  |  |  |
| <60 | 247 | 1.00 | 0.75 (0.33-1.70) | 0.69 (0.30-1.59) | 0.78 (0.36-1.70) | 0.58 | 0.22 |
| ≥60 | 225 | 1.00 | 0.48 (0.21-1.09) | 0.62 (0.27-1.41) | 1.23 (0.56-2.70) | 0.36 |  |
| BMI^a^, kg/m^2^ |  |  |  |  |  |  |  |
| <23 | 211 | 1.00 | 0.53 (0.21-1.29) | 0.69 (0.29-1.63) | 1.02 (0.47-2.24) | 0.69 | 0.82 |
| ≥23 | 361 | 1.00 | 0.64 (0.30-1.35) | 0.66 (0.30-1.46) | 0.92 (0.43-1.97) | 0.99 |  |
| Alcohol consumption^a^ |  |  |  |  |  |  |  |
| Never | 376 | 1.00 | 0.92 (0.50-1.72) | 0.75 (0.40-1.42) | 1.27 (0.70-2.33) | 0.48 | 0.19 |
| Weekly or daily | 96 | 1.00 | 0.08 (0.02-0.43) | 0.20 (0.04-1.05) | 0.20 (0.04-0.97) | 0.32 |  |
| Physical activity^a^ |  |  |  |  |  |  |  |
| <0.5 hours/week | 366 | 1.00 | 0.73 (0.39-1.40) | 0.71 (0.37-1.36) | 1.02 (0.55-1.88) | 0.81 | 0.89 |
| ≥0.5 hours/week | 100 | 1.00 | 0.33 (0.10-1.15) | 0.43 (0.12-1.52) | 0.89 (0.26-2.99) | 0.92 |  |
| Fasting status^a^ |  |  |  |  |  |  |  |
| Fasting | 139 | 1.00 | 0.57 (0.19-1.74) | 0.72 (0.26-2.06) | 0.94 (0.34-2.64) | 0.98 | 0.98 |
| Non-fasting | 333 | 1.00 | 0.64 (0.33-1.24) | 0.57 (0.28-1.15) | 1.00 (0.52-1.92) | 0.81 |  |
| Ferritin^a^, µg/mL |  |  |  |  |  |  |  |
| <204 | 216 | 1.00 | 0.66 (0.29-1.52) | 0.83 (0.35-1.98) | 0.59 (0.23-1.53) | 0.37 | 0.69 |
| ≥204 | 219 | 1.00 | 0.51 (0.20-1.30) | 0.29 (0.11-0.75) | 0.61 (0.26-1.44) | 0.41 |  |
| Hs-CRP^a^, mg/L |  |  |  |  |  |  |  |
| <1.3 | 229 | 1.00 | 0.96 (0.43-2.17) | 0.69 (0.30-1.62) | 1.20 (0.54-2.65) | 0.75 | 0.72 |
| ≥1.3 | 243 | 1.00 | 0.40 (0.17-0.92) | 0.53 (0.23-1.22) | 0.85 (0.39-1.86) | 0.92 |  |
| Adiponectin^a^, µg/mL |  |  |  |  |  |  |  |
| <6.65 | 236 | 1.00 | 0.77 (0.33-1.81) | 0.71 (0.29-1.75) | 0.67 (0.29-1.51) | 0.36 | 0.20 |
| ≥6.65 | 236 | 1.00 | 0.46 (0.20-1.06) | 0.57 (0.25-1.30) | 1.20 (0.54-2.67) | 0.52 |  |
| ALT^a^, IU/L |  |  |  |  |  |  |  |
| <21.5 | 230 | 1.00 | 0.76 (0.34-1.72) | 0.66 (0.29-1.54) | 1.52 (0.68-3.39) | 0.33 | 0.08 |
| ≥21.5 | 242 | 1.00 | 0.42 (0.18-0.99) | 0.46 (0.19-1.10) | 0.51 (0.23-1.14) | 0.25 |  |
| TG^a^, mmol/L |  |  |  |  |  |  |  |
| <1.91 | 231 | 1.00 | 0.33 (0.14-0.75) | 0.37 (0.15-0.88) | 1.10 (0.47-2.60) | 0.99 | 0.58 |
| ≥1.91 | 241 | 1.00 | 1.09 (0.45-2.61) | 0.86 (0.37-2.00) | 0.99 (0.45-2.17) | 0.88 |  |
| HDL-C^a^, mmol/L |  |  |  |  |  |  |  |
| <1.01 | 241 | 1.00 | 1.01 (0.46-2.19) | 0.86 (0.37-1.99) | 0.84 (0.39-1.79) | 0.58 | 0.36 |
| ≥1.01 | 231 | 1.00 | 0.37 (0.15-0.91) | 0.56 (0.24-1.28) | 1.20 (0.53-2.69) | 0.45 |  |
| HbA1c^b^, % |  |  |  |  |  |  |  |
| <6.5 | 256 | 1.00 | 0.81 (0.31-2.09) | 0.81 (0.31-2.14) | 1.26 (0.45-3.58) | 0.56 | 0.64 |
| ≥6.5 | 216 | 1.00 | 0.37 (0.12-1.16) | 0.42 (0.14-1.25) | 0.77 (0.28-2.12) | 0.99 |  |

^a^Odds ratio was estimated using unconditional logistic regression model adjusted for age at blood taken (continuous), dialect group (Cantonese, Hokkien), smoking status (never, past, and current smoker), alcohol intake (never, weekly or daily), weekly moderate-to-vigorous activity levels (<0.5, 0.5-3.9, and ≥4.0 hours/week), education level (primary school and below, secondary or above), history of hypertension (yes, no), fasting status (yes, no), body mass index (continuous), and menopausal status (premenopausal, postmenopausal).

^b^Odds ratio was estimated using conditional logistic regression model adjusted for abovementioned variables except for dialect group (Cantonese, Hokkien).

**Abbreviations:** ALT, alanine aminotransferase; BMI, body mass index; HbA1c, Hemoglobin A1c; HDL-C, high-density lipoprotein cholesterol; hs-CRP, high-sensitivity C-reactive protein; RBP4, retinol binding protein 4; TG, triglycerides.

**Supplemental Table S3.** Prospective studies of RBP4 and incident type 2 diabetes

| **Reference, Year** | **Study population** | **Study design** | **RBP4 assay** | **Diabetes ascertainment** | **Comparison** | **Adjustment of covariates** |
| --- | --- | --- | --- | --- | --- | --- |
| Luft et al, 2013 | The Atherosclerosis Risk in Communities (ARIC) Study;  N=1090; 543 cases  Age: 49-58 years (median: 53)  13% African American men,  35% African American women,  36% white men,  26% white women | Case-cohort study (9-year follow-up) | Plasma RBP4 measured from fasting samples  Assay: ELISA | Self-report of physician diagnosis, use of antidiabetic medications, or a fasting glucose value ≥7 mmol/L at 3 follow-up visits | Tertile 3 vs. 1 | Age, ethnicity, study center, ethanol intake, smoking, BMI, waist-to-hip ratio, glomerular filtration rate, inflammation score, adiponectin, leptin, TG, HDL-cholesterol, non-esterified fatty acids, hypertension and family history of diabetes |
| Sun et al, 2014 | The Nutrition and Health of Aging Population Study;  N=2091, 507 cases  Age: 50-70 years (mean: 58)  59% women, 41% men  Chinese | Prospective study (6-year follow-up) | Plasma RBP4 measured from fasting samples  Assay: ELISA | Self-report of physician diagnosis or taking anti-diabetic medications or a fasting glucose level ≥7 mmol/L | Quartile 4 vs. 1  &  Quintile 5 vs. 1 | Age, sex, region, residence, education, current smoking and drinking status, physical activity, family history of diabetes, BMI, CRP, adiponectin, TGs, HDL cholesterol, GGT, creatinine, HOMA-IR and hypertension |
| Current study | Singapore Chinese Health Study;  N=1142, 571 cases  Age: 50-76 years (mean: 60)  59% women, 41% men  Chinese | Nested case-control study (4-year follow-up) | Plasma RBP4 measured from mostly non-fasting samples  Assay: ELISA | Self-report of physician diagnosis | Quartile 4 vs. 1 | Age, smoking status, alcohol intake, weekly moderate-to-vigorous activity levels, education level, fasting status, BMI, hs-CRP, TG, HDL-cholesterol and ALT |

**Abbreviations:** ALT, alanine aminotransferase; BMI, body mass index; DNL, de novo lipogenesis; FA, fatty acids; GGT, Gamma-glutamyl transferase; HDL, high-density lipoprotein; hs-CRP, high-sensitivity C-reactive protein; TG, triglycerides.

**Supplemental Fig. S1.** Flowchart of the Singapore Chinese Health Study


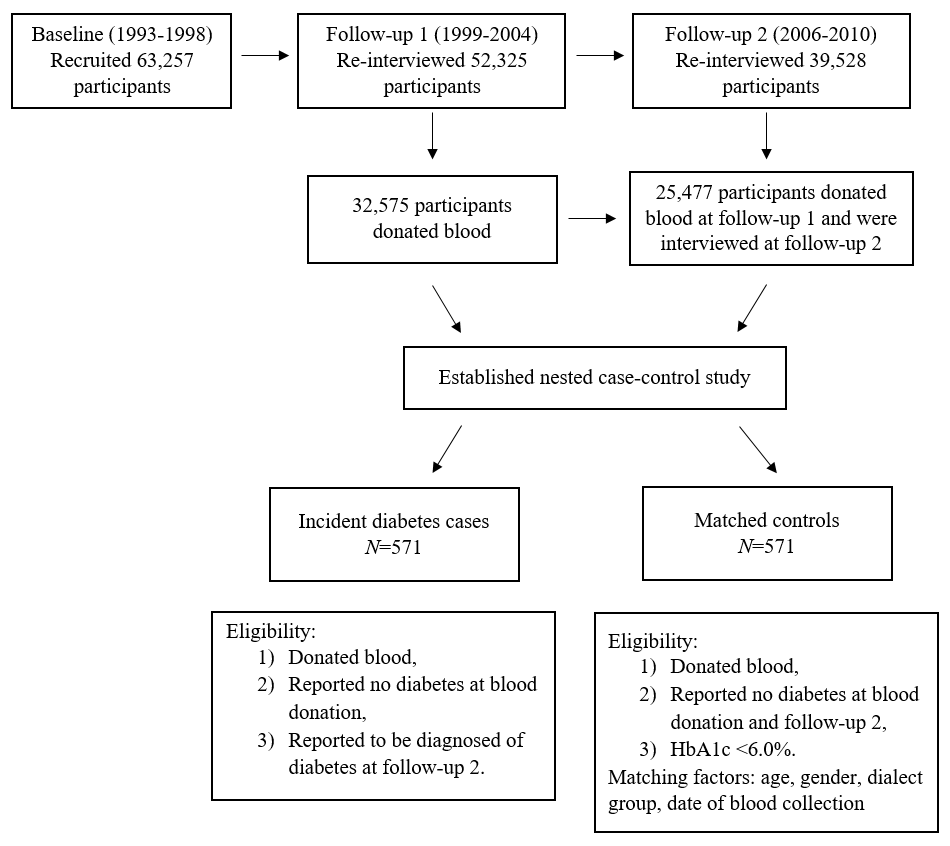


**Supplemental Fig. S2.** Spline analysis of the association between plasma levels of RBP4 and incident type 2 diabetes in women (A) and men (B)

*
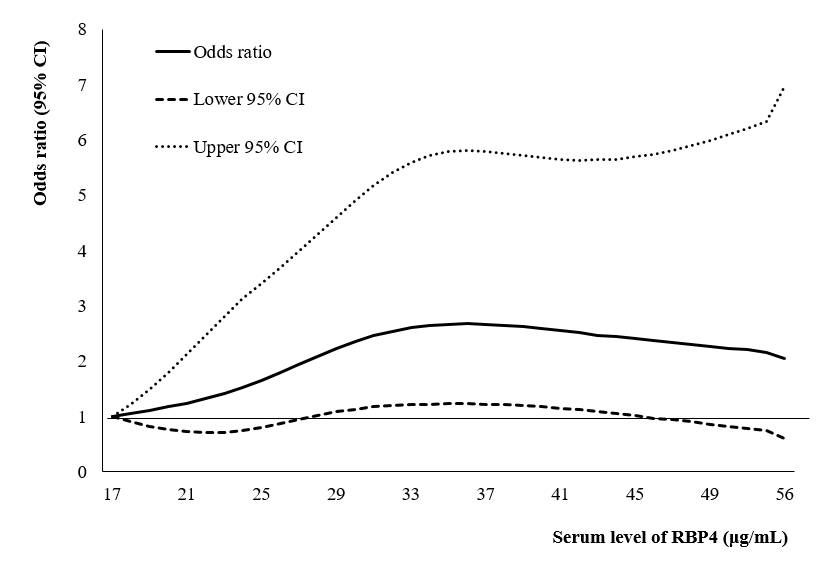
*

A

*
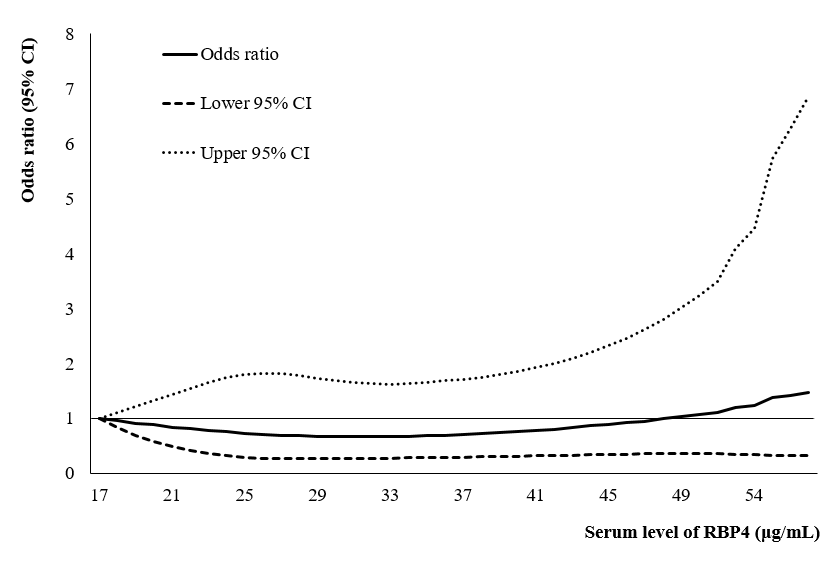
*

B

**Legend:** The *Solid line* represents point estimates of relative risk for the association between original value of RBP4 and incident type 2 diabetes, and the *dotted lines* represent the upper and lower bound of 95% CIs. Study participants with the lowest and highest 1% of RBP4 were excluded to minimize the potential impact of outliers. Cubic spline analysis was used to examine the association between RBP4 concentrations and risk of developing type 2 diabetes of model 2. *P*-nonlinearity =0.18 in women (A) *P*-nonlinearity =0.40 in men (B) in the cubic spline regression model.
